# Feasibility of implementing patient-reported outcome measures into routine breast cancer care delivery using a novel collection and reporting platform

**DOI:** 10.1093/jamiaopen/ooad108

**Published:** 2023-12-26

**Authors:** Elena Tsangaris, Colby Hyland, George Liang, Joanna O’Gorman, Dany Thorpe Huerta, Ellen Kim, Maria Edelen, Andrea Pusic

**Affiliations:** Division of Plastic and Reconstructive Surgery, Department of Surgery, Brigham and Women’s Hospital, Boston, MA 02115, United States; Division of Plastic and Reconstructive Surgery, Department of Surgery, Brigham and Women’s Hospital, Boston, MA 02115, United States; Division of Plastic and Reconstructive Surgery, Department of Surgery, Brigham and Women’s Hospital, Boston, MA 02115, United States; Division of Plastic and Reconstructive Surgery, Department of Surgery, Brigham and Women’s Hospital, Boston, MA 02115, United States; Division of Plastic and Reconstructive Surgery, Department of Surgery, Brigham and Women’s Hospital, Boston, MA 02115, United States; Division of Plastic and Reconstructive Surgery, Department of Surgery, Brigham and Women’s Hospital, Boston, MA 02115, United States; Division of Plastic and Reconstructive Surgery, Department of Surgery, Brigham and Women’s Hospital, Boston, MA 02115, United States; Division of Plastic and Reconstructive Surgery, Department of Surgery, Brigham and Women’s Hospital, Boston, MA 02115, United States

**Keywords:** improve, breast cancer, implementation, patient-reported outcomes, clinical care

## Abstract

**Objectives:**

imPROVE is a new Health Information Technology platform that enables systematic patient-reported outcome measure (PROM) collection through a mobile phone application. The purpose of this study is to describe our initial experience and approach to implementing imPROVE among breast cancer patients treated in breast and plastic surgery clinics.

**Materials and Methods:**

We describe our initial implementation in 4 phases between June 2021 and February 2022: preimplementation, followed by 3 consecutive implementation periods (P1, P2, P3). The Standards for Reporting Implementation Studies statement guided this study. Iterative Plan-Do-Study-Act (PDSA) cycles supported implementation, and success was evaluated using the Reach, Effectiveness, Adoption, Implementation, and Maintenance framework.

**Results:**

Qualitative interviews conducted during the preimplementation phase elicited 4 perceived implementation barriers. Further feedback collected during each phase of implementation resulted in the development of brochures, posters in clinic spaces, and scripts for clinic staff to streamline discussions with patients, and the resolution of technical issues concerning patient login capabilities, such as compatibility with cell phone software and barriers to downloading imPROVE. Feedback also generated ideas for facilitating provider interpretation of PROM results. By the end of P3, 2961 patients were eligible, 1375 (46.4%) downloaded imPROVE, and 1070 (36.1% of those eligible, 78% of those who downloaded) completed at least 1 PROM.

**Discussion and Conclusion:**

Implementation efforts across 2 surgical departments at 2 academic teaching hospitals enabled collaboration across clinical specialties and longitudinal PROM reporting for patients receiving breast cancer care; the implementation effort also highlighted patient difficulties with mobile app-based PROM collection, particularly around initial engagement.

## Introduction

Improving healthcare quality depends on the clinician’s ability to address concerns that are most important to patients. While metrics such as morbidity and mortality have traditionally taken precedence in evaluating patient outcomes, there has been an increasing emphasis on obtaining information directly from patients related to their quality of life (QOL). Current standards for physician-patient encounters and medical tests do not consistently evaluate nor do they address QOL outcomes, with physicians often underestimating the severity of symptoms.^1^

Patient-reported outcome measures (PROMs) enable clinicians to understand QOL outcomes, such as physical and psychosocial well-being and satisfaction with care, from the patient’s perspective.^2^ While PROMs have traditionally been used in research,^3^ their use in routine clinical care enhances the quality of care delivered to patients, clinician–patient interactions, and patient outcomes.^3–10^ In addition, routine PROM collection enables more accurate symptom measurement.^11–18^ For this reason, there is increasing interest in using PROMs to inform care delivery.^19^

Despite the appeal of incorporating PROM collection in routine clinical care, several challenges prevail, limiting their uptake and use. First, the perceived value of PROMs is not unanimous. Negative perceptions of PROMs and the lack of knowledge about their positive impact on care delivery can impede their successful uptake and use. Second, there is no transferrable tool available to support real-time collection, storing, analyzing, or reporting PROMs.^20^^,^^21^ Third, existing platforms and electronic health records (EHR) currently supporting the collection of PROMs are widely underutilized due to their inability to meet the providers’ information needs and poor integration and synchronization with the EHR.^22^ Finally, successful and sustainable PROM implementation initiatives have been difficult to achieve.^22^ Some studies report a lack of time to appropriately integrate PROMs into clinical practice as a barrier to their use.^22^

In recognizing the value of PROM collection in routine clinical care, our team recently developed a new Health Information Technology (HIT) platform called imPROVE, which enables the systematic collection of PROMs through a mobile phone application (app).^23^ imPROVE was designed and developed using best practices in user-centered design, agile development, and qualitative methods described in detail elsewhere.^23^ In this article, we describe our initial experience and approach to implementing imPROVE among patients in breast and plastic surgery clinics over 9 months. We present recruitment and completion rates and key barriers and facilitators to implementation.

## Methods

### imPROVE

imPROVE is a mobile application wherein patients can complete PROMs, view their results over time, and receive links to tailored resources (support groups, informational videos about surgery, etc.). imPROVE also has a clinician dashboard component wherein providers can view individual patient profiles with their PROM data displays and links to patient resources.^23^ imPROVE incorporates the breast cancer standard outcome set of the International Consortium for Health Outcomes Measurement (ICHOM)^24^^,^^25^ and the complete BREAST-Q Breast Cancer Module.^26^ Algorithms for PROM scoring are embedded within the application. PROMs are administered preoperatively and postoperatively at 2 weeks, 6 weeks, 3 months, and every 6 months thereafter, as per ICHOM recommendations.^24^^,^^25^ Patients can also complete PROMs at any time on demand.

### Implementation and study design

The Standards for Reporting Implementation (StaRI) Studies statement^27^ guided this study. We describe our initial implementation in 4 phases: preimplementation, and 3 consecutive implementation periods (P1, P2, P3) between June 2021 and February 2022, and each implementation period spanned 3 months. We implemented imPROVE in breast and plastic surgery clinics at 2 academic hospitals (Dana-Farber Cancer Institute [DFCI] and Brigham and Women’s [BWH]), consisting of 4 separate sites.

During the preimplementation phase, a trained qualitative researcher conducted 1-h qualitative interviews with a breast surgeon, administrative assistant, nurse practitioner, and a plastic surgery research staff member involved in prior PROM implementation initiatives. The interviewer used a semi-structured guide to probe each participant about clinic workflows, their perceived value of PROMs, and potential barriers to PROM implementation. Interviews were audiorecorded, transcribed verbatim, and coded inductively within Microsoft Word (2020) using top-level domains and themes. A qualitative description was used to guide the analysis.^28^^,^^29^

We used findings from the preimplementation phase to design our initial implementation strategy. Implementation was supported by iterative Plan-Do-Study-Act (PDSA) cycles,^30^ involving weekly (plastic surgery) and biweekly (breast surgery) meetings led by the imPROVE project manager. The meetings consisted of the core project team (including information technology support that maintained active communication with the software vendor), breast and plastic surgery “clinic champions” (dedicated staff who raised awareness and served as points of contact in clinics), and research assistants. Within meetings, we reviewed enrollment and PROM completion trends, discussed implementation challenges, and planned strategies for the coming week(s). We developed tableau dashboards to monitor implementation success, including data displays of patient enrollment, activation, and PROM completion rates.

The Reach, Effectiveness, Adoption, Implementation, and Maintenance (RE-AIM) framework^31^^,^^32^ guided the evaluation of implementation success, defined as obtaining activation and PROM completion rates of 70% or higher. For the present study, reach, effectiveness, and adoption were evaluated (Table 1).

**Table 1.** Primary outcomes to evaluate reach, effectiveness, and adoption.

RE-AIM domain	Outcomes
Reach	Clinical and demographic comparisons of eligible patients who downloaded imPROVE versus those who did not % Downloaded/eligible % Completed PROMs at any timepoint/eligible
Effectiveness	Feedback reports from clinic staff
Adoption	% Downloaded patients completing PROMs at least once
	% Eligible patients downloading imPROVE by care team
	% Downloaded patients completing PROMs by clinician

This study was a quality improvement initiative at BWH and DFCI and was not formally supervised by their Institutional Review Board, per their policies. No formal consent was obtained; patients were informed of this new program as part of routine clinical care and had the option to decline enrollment without any impact on the quality of care received.

### Study participants

All patients receiving care for breast cancer in one of the participating clinics over the age of 40 were eligible for imPROVE, regardless of treatment status (ie, pre/post-operative or long-term follow-up). A parallel research study at our institution precluded participation for participants under the age of 40. Patients who were nonsurgical candidates had poor English proficiency, cognitive impairment, or a health condition preventing their ability to use a mobile application, had no smartphone access, or had poor proficiency in mobile app use/download (ie, trouble downloading applications or navigating technology) were excluded. The study team screened patients for eligibility using EHR reports and sent eligible patients a text message invitation to download imPROVE and complete an assessment (ie, their PROMs) before their clinic visit.

At clinic visits, staff (medical and administrative assistants) and clinicians (physician assistants, registered nurses, and breast and plastic surgeons) reminded patients about imPROVE—assisted by handout brochures and posters hung in communal areas.

### Data analysis

We used descriptive statistics to describe the imPROVE cohort. We compared patient disease and treatment characteristics between active and pending groups using the Chi-square test or Fisher exact test for categorical variables and the independent Student’s *t*-test for continuous variables. Before statistical tests were conducted, Kurtosis and Skewness were used to assess the normality of the data,^33^ and nonparametric tests were applied when values exceeded +2.0.^34^*P*-values≤.05 were considered statistically significant. Statistical tests were performed in SPSS version 26.0 (IBM Corporation, Armonk NY, USA for Windows/Apple Mac).

## Results

### Preimplementation

Qualitative interviews conducted during the preimplementation phase elicited the following perceived implementation barriers: (1) clinic workflow and wait times, (2) compliance from patients and clinic staff, (3) data access and visualization, and (4) education and training of clinic staff. Representative statements for each barrier are listed in Table 2. We used these findings to guide the PDSA cycles in the subsequent implementation periods.

**Table 2.** Qualitative barriers to PROM implementation.

Barrier	Representative statement
Clinic workflow and wait times	“it’s just that the time of arrival is variable, and then the room crunch is the big thing that when you are in a room, you basically are only allowed to have 1, possibly 2 rooms at any given time and you have a very full schedule and like once that patient is done they really need to leave that room or it backs up everything”
Compliance from patients and staff	“I worry that patients will not want to fill it out if they feel that their surgeon is not even looking at it”
Data access and visualization	“I think the other piece is the ability to actually use the data with the patient which we really can’t in this current form um and the ability to get the data out”
Education and training of clinic staff	“I think one of the other big challenges is that um the front desk staff is very willing, but this is very new to them they don’t totally know what they are doing, and it wasn’t going off without a hitch, um and because of that they were really looking for support if we are going to be doing it”

### P1, June to August 2021

Our team first implemented imPROVE in 1 surgeon’s clinic (plastic surgery) at the start of P1 to intensively study and optimize integration in the clinic and ease adoption by clinic staff before expanding out to additional clinics. During this time, a dedicated research assistant supported implementation efforts in the clinic. Throughout this period, daily feedback reports from clinic staff conveyed concerns about the time needed to explain imPROVE to patients, low levels of clinic staff awareness of imPROVE and its functionality, and insufficient education or supporting materials to support clinic staff discussions with patients. To address these concerns, we developed brochures to serve as an adjunct for clinic staff in discussing imPROVE with patients and as an additional informational resource. Our team also developed and hung posters in clinic spaces and disseminated scripts to clinic staff to streamline the discussion of imPROVE with patients.

**Figure 1.**
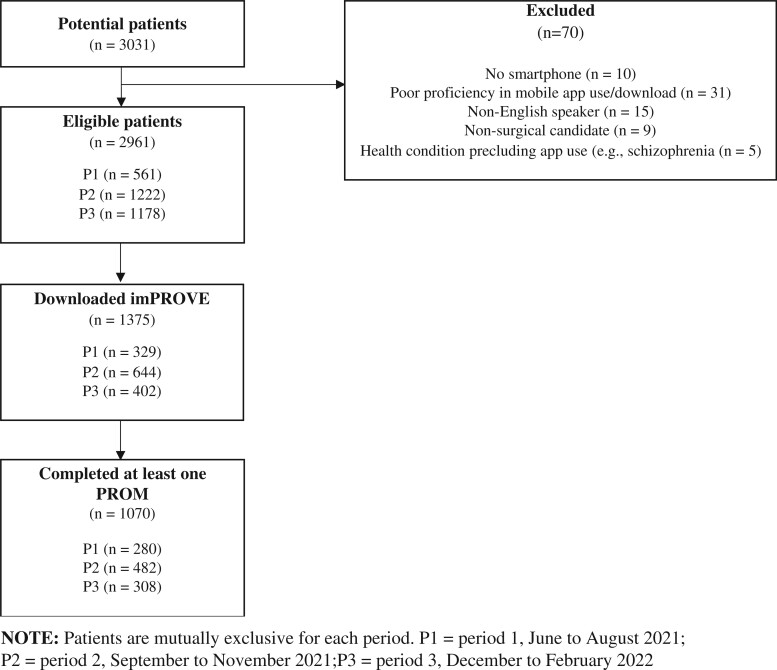
Patient enrollment from June 2021 to February 2022.

By the end of P1, 7 plastic surgeons and 1 breast surgeon with their respective care teams were enrolling patients in imPROVE. Of the 561 eligible patients, 329 (59%) downloaded imPROVE, and 280 (50% of those eligible and 78% of those who downloaded) completed at least 1 PROM (Figure 1). Among patients who downloaded imPROVE, on average, it took 19 days (range 0-161) for patients to download after they were first notified.

### P2, September to November 2021

**Figure 2.**
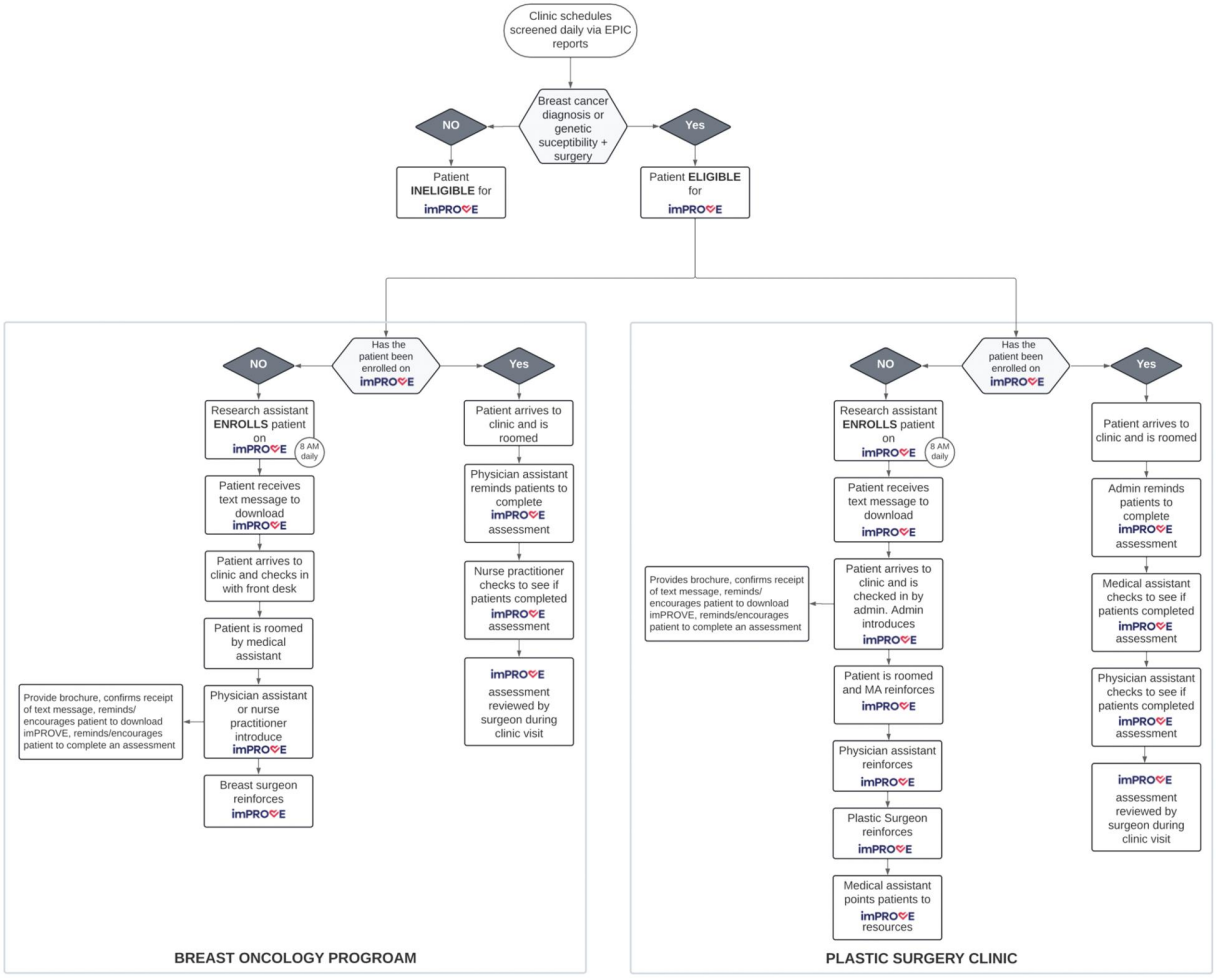
Recruitment and clinic workflows. *Note:* Patients are mutually exclusive for each period. P1, period 1, June to August 2021; P2, period 2, September to November 2021; P3, period 3, December to February 2022.

During phase 2, 1 additional plastic surgeon and 3 additional breast surgeons began enrolling patients in imPROVE. During this phase, clinic staff feedback mainly involved technical issues concerning patient login capabilities, such as compatibility with cell phone software and barriers to downloading imPROVE (eg, patients forgetting passwords). To address these concerns, we established active troubleshooting and documentation of technical issues, which facilitated streamlined communication with the software vendor to address any problems. In addition, PDSA feedback informed changes to workflows which needed to be optimized and tailored to the unique clinic designs and staffing structures of breast and plastic surgery clinics (Figure 2). The project team began sending text message reminders to eligible patients who had not yet downloaded imPROVE after their initial encounter to increase download rates.

Of the 1222 eligible patients, 644 (53%) downloaded imPROVE, and 482 (39% of those eligible, 75% of those who downloaded) completed at least 1 PROM. Among patients who downloaded imPROVE, it took 7 days on average (range 0-97) for patients to download after initial notification (Figure 1).

### P3, December 2021 to February 2022

By the end of P3, all breast (*n* = 11) and plastic (*n* = 8) surgeons at the 4 clinic locations were enrolling patients in imPROVE. Feedback during this period mainly involved ideas for facilitating provider interpretation of PROM data both in terms of optimal provider-facing dashboards (desktop, tablet, or mobile phone) and contextualizing results within clinical care and available supportive resources. To address this, our team created monthly enrollment and feedback reports for clinic staff and developed instructions for providers regarding access to PROM results on desktop dashboards and mobile phones. Clinic “champions” routinely encouraged faculty to discuss PROMs as critical assessments for clinical care rather than research tools and to elicit and discuss PROM data at each clinical encounter with patients.

Of the 1178 eligible patients, 402 (34%) downloaded imPROVE, and 308 (26% of those eligible, 77% of those who downloaded) completed at least 1 PROM. Among patients who downloaded imPROVE, on average, it took 1 day (range 0-28) for patients to download after being notified (Figure 1).

### Overall implementation and patient characteristics, June 2021 to February 2022

**Figure 3.**
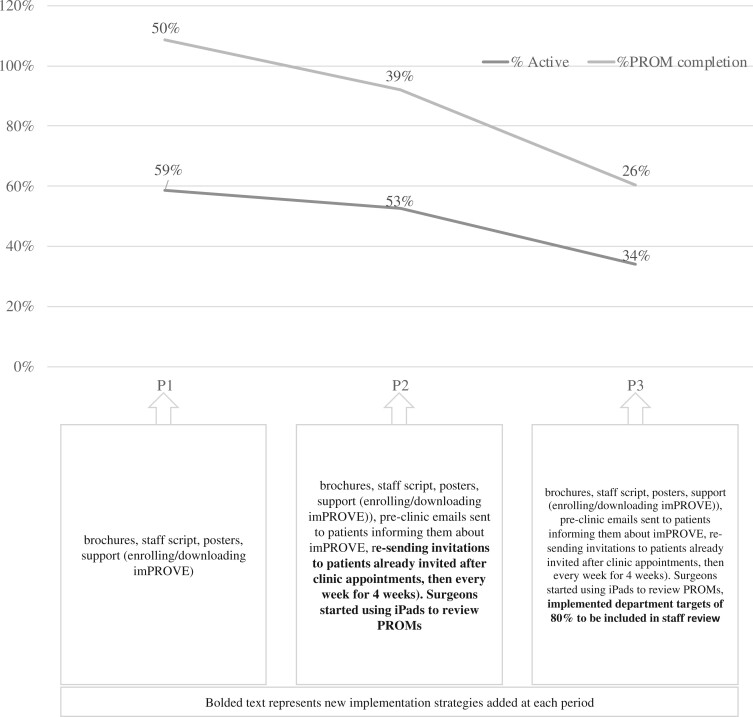
Run cart of the number of patients who downloaded imPROVE and completed a PROM from P1 through P3.

Table 3 demonstrates the clinical and demographic characteristics of patients throughout implementation. Between June 2021 and February 2022, 3031 patients were identified as candidates for imPROVE. Of these, 2961 were eligible, 1375 (46.4%) downloaded imPROVE, and 1070 (36.1% of those eligible, 78% of those who downloaded) completed at least 1 PROM (Figure 1). Figure 3 provides a run cart that outlines the number of patients who downloaded imPROVE and those who completed a PROM from P1 through P3.

**Table 3.** Clinical and demographics information of eligible patients (total = 2961; P1 = 561; P2 = 1222; P3 = 1178) invited to imPROVE.

		Total	P1	P2	P3
		*N* (%)	*N* (%)	*N* (%)	*N* (%)
Age (years)	≤49	781 (26.4)	182 (32.4)	309 (25.3)	290 (24.6)
	50-59	952 (32.2)	206 (36.7)	403 (33.0)	343 (29.1)
	60-69	739 (25.0)	119 (21.2)	306 (25.0)	314 (26.7)
	≥70	489 (16.5)	54 (9.6)	204 (16.7)	231 (19.6)
Race	White	2481 (83.8)	487 (86.8)	1034 (84.6)	960 (81.5)
	Black or African American	127 (4.3)	23 (4.1)	50 (4.1)	54 (4.6)
	Asian	105 (3.5)	16 (2.9)	35 (2.9)	54 (4.6)
	Other	68 (2.3)	9 (1.6)	35 (2.9)	24 (2.0)
	Prefer not to say	43 (1.5)	5 (0.9)	19 (1.6)	19 (1.6)
	Missing	137 (4.6)	21 (3.7)	49 (4.0)	67 (5.7)
Ethnicity	Hispanic	101 (3.4)	21 (3.7)	48 (3.9)	32 (2.7)
	Non-Hispanic	2613 (88.2)	503 (89.7)	1076 (88.1)	1034 (87.8)
	Prefer not to say	24 (0.8)	4 (0.7)	12 (1.0)	8 (0.7)
	Missing	223 (7.5)	33 (5.9)	86 (7.0)	104 (8.8)
Education completed	Less than high school	21 (0.7)	5 (0.9)	8 (0.7)	8 (0.7)
	High school	530 (17.9)	58 (10.3)	241 (19.7)	231 (19.6)
	College/trade/university	1464 (49.4)	204 (36.4)	659 (53.9)	601 (51.0)
	Graduate school	213 (7.2)	28 (5.0)	89 (7.3)	96 (8.1)
	Post-graduate school	227 (7.7)	17 (3.0)	99 (8.1)	111 (9.4)
	Prefer not to say	64 (2.2)	5 (0.9)	34 (2.8)	25 (2.1)
	Other	42 (1.4)	4 (0.7)	23 (1.9)	15 (1.3)
	Missing	400 (13.5)	239 (42.6)	75 (6.1)	86 (7.3)
Marital status	Single	433 (14.6)	75 (13.4)	176 (14.4)	182 (15.4)
	Married/common law	1965 (66.4)	383 (68.3)	808 (66.1)	774 (65.7)
	Separated/divorced/widowed	433 (14.6)	87 (15.5)	181 (14.8)	165 (14.0)
	Prefer not to say	4 (0.1)	0 (0.0)	3 (0.2)	1 (0.1)
	Missing	126 (4.3)	16 (2.9)	54 (4.4)	56 (4.8)
Type of surgery	Planning	531 (17.9)	56 (10.0)	218 (17.8)	257 (21.8)
	Lumpectomy	953 (32.2)	80 (14.3)	337 (27.6)	536 (45.5)
	Mastectomy	215 (7.3)	45 (8.0)	78 (6.4)	92 (7.8)
	Reconstruction	1262 (42.6)	380 (67.7)	589 (48.2)	293 (24.9)
Laterality	Unilateral	241 (8.1)	114 (20.3)	41 (3.4)	86 (7.3)
	Bilateral	2073 (70.0)	384 (68.4)	921 (75.4)	768 (65.2)
	Missing	116 (3.9)	7 (1.2)	42 (3.4)	67 (5.7)
	Not applicable	531 (17.9)	56 (10.0)	218 (17.8)	257 (21.8)

The mean age of eligible patients was 58 years (SD = 11; range = 40-94). Among those who did and did not download imPROVE, the mean age was 56 (SD = 10) and 60 (SD = 11), respectively. When comparing patients who did or did not download imPROVE, significant differences were identified by age group (*P* <.001), marital status (*P* =.004), surgery type (*P* <.001), and laterality (*P* <.001) (see Table 4), with patients aged up to 59 years who were married, had reconstruction surgery and had bilateral breast cancer more likely to download imPROVE.

**Table 4.** Clinical and demographics information of patients who did/did not download imPROVE.

		Did not download imPROVE (*N* = 1586)		Downloaded imPROVE (*N* = 1375)		*P*
		*N*	%	*N*	%	
Age (years)	<49	352	22	429	31	<.001
	50-59	491	31	461	34	
	60-69	398	25	341	25	
	≥70	345	22	144	10	
Race	White	1314	83	1167	85	=.513
	Black or African American	77	5	50	4	
	Asian	54	3	51	4	
	Other	36	2	32	2	
	Missing	105	7	75	5	
Ethnicity	Hispanic	55	3	46	3	=.809
	Non-Hispanic	1391	88	1222	89	
	Missing	140	9	107	8	
Education completed	Less than high school	12	1	9	1	=.080
	High school	281	18	249	18	
	College/trade/university	694	44	770	56	
	Graduate school	113	7	100	7	
	Post-graduate school	122	8	105	8	
	Other	17	1	25	2	
	Missing	347	22	117	9	
Marital status	Single	245	15	188	14	=.004
	Married/common law	1018	64	947	69	
	Separated/divorced/widowed	260	16	173	13	
	Missing	63	4	67	5	
Type of surgery	Planning	354	22	177	13	<.001
	Lumpectomy	547	34	406	30	
	Mastectomy	134	8	81	6	
	Reconstruction	551	35	711	52	
Laterality	Unilateral	185	12	56	4	<.001
	Bilateral	1000	63	1073	78	
	Missing	47	3	69	5	
	Not applicable	354	22	177	13	

Similar results were evidenced when comparing patients who downloaded imPROVE and those who did or did not complete a PROM. Significant differences were evident by age group (*P* <.001) and type of surgery (*P* <.001), with patients aged up to 59 years who had a reconstruction surgery more likely to complete a PROM. Table 5 demonstrates the clinical and demographic characteristics of patients who downloaded and completed at least 1 PROM (vs those who did not).

**Table 5.** Clinical and demographics information of patients who downloaded imPROVE and did/did not complete at least 1 PROM.

		Downloaded imPROVE and did not complete a PROM (*N* = 334)		Downloaded imPROVE and completed at least 1 PROM (*N* = 1041)		*P*
		*N*	%	*N*	%	
Age (years)	<49	68	20	361	35	<.001
	50-59	125	37	336	32	
	60-69	86	26	255	24	
	≥70	55	16	89	9	
Race	White	282	84	885	85	=.948
	Black or African American	13	4	37	4	
	Asian	12	4	39	4	
	Other	9	3	23	2	
	Missing	18	5	57	5	
Ethnicity	Hispanic	6	2	40	4	=.077
	Non-Hispanic	298	89	924	89	
	Missing	30	9	77	7	
Education completed	Less than high school	3	1	6	1	=.591
	High school	62	19	187	18	
	College/trade/university	191	57	579	56	
	Graduate school	21	6	79	8	
	Post-graduate school	20	6	85	8	
	Other	7	2	18	2	
	Missing	30	9	87	8	
Marital status	Single	43	13	145	14	=.780
	Married/common law	228	68	719	69	
	Separated/divorced/widowed	45	13	128	12	
	Missing	18	5	49	5	
Type of surgery	Planning	53	16	124	12	<.001
	Lumpectomy	121	36	285	27	
	Mastectomy	17	5	64	6	
	Reconstruction	143	43	568	55	
Laterality	Unilateral	14	4	42	4	=.682
	Bilateral	243	73	830	80	
	Missing	24	7	45	4	
	Not applicable	53	16	124	12	

Overall, 19 care teams, consisting of a plastic or breast surgeon, and their respective staff (administrative, medical and physician assistants, or registered nurses), implemented imPROVE during the study period.

## Discussion

This article describes our initial experience implementing imPROVE, a new HIT platform for PROM collection among surgical breast cancer patients in breast and plastic surgery clinics over 9 months. We employed iterative PDSA cycles to address the many and varied barriers to implementation that changed throughout the study period, ultimately refining the implementation of systematic PROM collection in clinical care across 19 clinics in 4 locations. These results inform existing and future implementation efforts for routine PROM collection in clinical care using smartphone-based HIT platforms.

Throughout the 9-month implementation period, PDSA cycles proved essential for elucidating and addressing the most salient barriers to implementation at a given time. Initial feedback broadly highlighted gaps in clinic staff knowledge of imPROVE and a need for more comfort with its functionality, which was addressed with informational brochures, posters, scripts for administrative staff, and ongoing in-person facilitation by research assistants. Subsequently, technical issues at the patient level became notable and were resolved with iterative problem-solving with the software vendor. In the latest phase (P3), feedback identified the need to facilitate provider-level engagement and application of PROs during a clinic encounter which we did with several added features. These findings suggest that, while optimization of technical issues and logistics are critical, successful implementation also depends on adequate staff onboarding and a culture shift in clinical operations, whereby routine PROM collection is recognized as a standard of care procedure among providers and staff. These findings are supported by research that has shown a lack of awareness of PROMs within the workplace to be among the most significant barriers to their implementation.^35^ In addition, these findings highlight the need for adaptable implementation strategies that can be iterated in real-time. As each issue was addressed, others emerged as most salient for further optimization.

While PDSA cycles guided day-to-day operations and long-term strategies, the RE-AIM framework allowed us to gauge implementation success over time. In pre-defining reach, effectiveness, and adoption goals (Table 1), for example, we were able to better focus our efforts across many different providers and clinical settings. This framework also allowed us to structure PDSA cycles around common goals and elucidate barriers, facilitators, and lessons learned for critical metrics of implementation success.

Regarding reach, we observed that the time between notification of imPROVE and subsequent download was shortened for each implementation period. This finding may be explained, in part, by the longer follow-up time of patients notified within the initial implementation periods, as these patients may have had multiple appointments and text message reminders to download imPROVE at later timepoints. It is also likely due to greater technology optimization, workflows, and communication throughout implementation. Nonetheless, we found routine reminders, both in the form of text messages to patients and from front desk staff at follow-up appointments, to be critical for the ongoing enrollment of patients.

Our study also revealed notable differences in clinical and demographic characteristics between patients who did and did not download imPROVE and between those who did and did not complete at least 1 PROM. Patients who did not download imPROVE were significantly older, unmarried, and had lower educational attainment than patients who downloaded improve and were more likely to have had a mastectomy and unilateral surgery. Similar differences were observed for age and type of surgery between patients who did and did not complete at least 1 PROM. Notably, however, there was no significant difference in race between those who did and did not download imPROVE and those who completed at least 1 PROM. Studies investigating PROM implementation in diverse and underrepresented patient populations have demonstrated disparate response rates, which may be partly explained by the unique concerns and preferences of these populations.^36^ A better understanding of the concerns of these patients will be the topic of future investigation to guide further implementation efforts and achieve equitable PROM collection.

The effectiveness of our implementation was primarily assessed through weekly clinic feedback obtained as part of our PDSA cycles. In our experience, optimizing clinic workflows, utilizing PROM “champions” in the clinic, and the cultural shift of using PROMs as a routine assessment rather than a research tool were critical for effective implementation. PROM implementation programs in routine clinical care have been noted previously to incur additional costs, with additional staffing resource requirements.^21^^,^^37^ Accordingly, our study highlighted the need for collaboration among providers, research assistants, technical support staff, and the data management team. In attempting to integrate imPROVE into standard clinical care workflows, for example, efforts were needed to ensure that all care team members were aligned. We identified care team members whose involvement was essential for implementation success. These included administrative assistants and front desk staff, who were often the patients’ first point of contact regarding the mobile app; medical assistants, who were able to confirm and encourage app usage during rooming and vital signs intake; physician assistants and physicians, who discussed app usage and individual PROM results during appointments; and research assistants, who provided valuable feedback on implementation efforts and individualized technical support to patients and providers while the mobile app was being optimized.

Concerning adoption, although most patients who downloaded imPROVE eventually completed at least 1 PROM, the primary attrition rate in our study among eligible patients was in the initial download step. Indeed, we observed that the overall percentage of patients who downloaded imPROVE declined over time as we expanded to more clinics and providers, despite ongoing optimization efforts and steady PROM completion rates among those who did download the app. Many patients reported not remembering their app store account password, which precluded imPROVE download. The decline in the overall percentage of patients who downloaded imPROVE may also be explained by fewer opportunities for follow-up and text message reminders for patients recruited in P3. But this barrier aside, our experience revealed that some patients might have been overwhelmed in the context of their clinic visit and preferred not to download the app. Further, some patients prefer to use their hospital patient portals and may have found the additional mobile application onerous.

Although routine PROM collection using mobile applications has been successful in other clinical settings,^33^^,^^38^ our results suggest that there may be better ways to engage with all patients other than mobile applications. Instead, mobile-friendly web-apps may enable greater integration with existing patient portals and EHR systems to reduce barriers to PROM completion and will be an important area of future study for our team.

There is a pressing need for acceptable and effective ways to collect PROMs in routine clinical care, to improve patient outcomes, enhance patient-provider communication, and inform quality improvement and reimbursement policies.^21^^,^^39^^,^^40^ Our study adds to the growing body of literature describing the implementation of PROMs in routine clinical care. Notably, our study details implementation efforts across 2 surgical departments at 2 academic teaching hospitals, which allowed for collaboration across clinical specialties and longitudinal PROM reporting for patients undergoing breast cancer care. The implementation effort highlighted patient difficulties with mobile app-based PROM collection, particularly around initial engagement.

There were limitations to our study. First, although research assistants were instrumental in supporting patients and providers during the initial stages of implementation, this was an additional resource needed for successful implementation. While the need for additional staffing and resources has been cited as a barrier to routine PROM collection,^21^ the level of support provided by research assistants in our study reassuringly lessened over time as technical components of the app were optimized to the point where certain providers no longer required support. Second, the imPROVE app was not integrated within the patient portal or EHR at the time of this study and was only accessible via smartphones, which may have served as a barrier to patient use. However, smartphone ownership has become increasingly prevalent among adults, and reliance on smartphones for internet access is particularly common among racial and ethnic minorities, low income, and low education adults.^41^ Third, this study was limited to adults aged 40 and older, which limited our results generalizability. Nonetheless, because smartphone ownership and mobile app usage are more common among younger adults,^41^^,^^42^ we believe implementation would be as much, if not more, successful in this younger population and will be an important focus of future research.

This study lays the groundwork for several critical next steps. Given our initial success in breast cancer and plastic surgery clinics at 2 large academic medical centers, we hope to expand these efforts to patients receiving care in community cancer centers. As PROM collection in routine clinical care becomes increasingly more common, it will be imperative to ensure that PROM data are being collected from all patients, especially those who have been historically excluded from research to date, such as racial and ethnic minorities and patients with low socioeconomic status.^43^^,^^44^ In addition, we will explore alternatives to third-party mobile-app-based PROM collection and to expand across medical specialties, departments, and institutions.

## Data Availability

The data underlying this article will be shared on reasonable request to the corresponding author.
